# A Novel Resveratrol Analog Upregulates SIRT1 Expression and Ameliorates Neointima Formation

**DOI:** 10.3389/fcvm.2021.756098

**Published:** 2021-11-02

**Authors:** Baohui Yuan, He Liu, Xiaoliang Dong, Xiaohua Pan, Xun Sun, Jia Sun, Li-Long Pan

**Affiliations:** ^1^Wuxi School of Medicine and School of Food Science and Technology, Jiangnan University, Wuxi, China; ^2^State Key Laboratory of Food Science and Technology, Jiangnan University, Wuxi, China; ^3^School of Pharmacy, Fudan University, Shanghai, China

**Keywords:** (*R*)-TML104, neointima formation, nicotinamide adenine dinucleotide phosphate oxidase 4, nuclear factor-κB, vascular smooth muscle cells, reactive oxygen species, SIRT1

## Abstract

Neointima formation is a serious complication caused by mechanical trauma to the vessel. (*R*)-4,6-dimethoxy-3-(4-methoxy phenyl)-2,3-dihydro-1H-indanone [(*R*)-TML 104] is a synthesized analog of the natural product resveratrol sesquiterpenes (±)-isopaucifloral F. The present study aimed to investigate the effects and underlying mechanisms of (*R*)-TML104 on neointima formation. Our results showed that (*R*)-TML104 prevented neointima formation based on a carotid artery injury model in mice. Furthermore, (*R*)-TML104 inhibited platelet-derived growth factor-BB (PDGF-BB)-induced vascular smooth muscle cells (VSMC) phenotypic transformation, evidenced by increased α-smooth muscle actin, reduced VSMC proliferation, and migration. Simultaneously, (*R*)-TML104 upregulated sirtuin-1 (SIRT1) expression in VSMC. We further uncovered that SIRT1 expression is critical for the inhibitory effects of (*R*)-TML104 on PDGF-BB-induced VSMC phenotypic transformation *in vitro* and injury-induced neointima formation *in vivo*. Finally, (*R*)-TML104-upregulated SIRT1 inhibited PDGF-BB-induced VSMC phenotypic transformation by downregulating nicotinamide adenine dinucleotide phosphate oxidase 4 expression via decreasing nuclear factor-κB acetylation. Taken together, these results revealed that (*R*)-TML104 upregulates SIRT1 expression and ameliorates neointima formation. Therefore, the application of (*R*)-TML104 may constitute an effective strategy to ameliorate neointima formation.

## Introduction

Cardiovascular diseases are the major cause of death worldwide (1). Although surgery is a commonly used strategy to treat cardiovascular disease, the surgical process may cause vascular inflammation, potentially leading to endothelial damage and subsequent neointima formation (2, 3). Neointima formation may result in vascular restenosis (2–4). The underlying mechanisms of neointima formation remain unclear. However, current medical therapies for inhibiting neointima formation are still scarce, making the development of novel strategies a necessity.

Phenotypic transformation of the vascular smooth muscle cells (VSMC) plays a vital role in neointima formation and can be triggered by oxidative stress, which stems from the excessive production of reactive oxygen species (ROS) (5, 6). The major source of ROS in VSMC is the nicotinamide adenine dinucleotide phosphate (NADPH) oxidase (NOX) family (7). Additionally, the NOX-derived ROS can be modulated by sirtuin-1 (SIRT1) (8, 9). Platelet-derived growth factor-BB (PDGF-BB) is a major driving factor of the VSMC phenotypic transformation involved in neointima formation (10). During neointima formation, vascular stabilizing factors are attenuated, including SIRT1, a NAD (+)-dependent histone deacetylase (11, 12). Meanwhile, accumulating evidence suggests that various transcription factors are regulated by SIRT1, including nuclear factor-κB (NF-κB) (13, 14). Additionally, NF-κB activation is a pathological hallmark of VSMC phenotypic transformation (15, 16). The NF-κB activity can be mediated by sirtuin-1 (SIRT1)-mediated deacetylation (11, 17). Therefore, upregulation of SIRT1 may be a potential strategy for inhibiting VSMC phenotypic transformation.

Resveratrol, an active polyphenol compound, is found in red wine, grapes, and peanuts, and exhibits antioxidant and anti-inflammatory effects (18, 19). Resveratrol has attracted massive attention for its health benefits, including its advantageous effects on vascular diseases (20–22). It had also been shown that the beneficial properties of resveratrol are involved in multiple signaling pathways and oxygen species genes (23, 24). Several studies have indicated that numerous resveratrol analogs have better effects than resveratrol on improving disease (25, 26). In this study, we evaluated the effect of (*R*)-4, 6-dimethoxy-3-(4-methoxy phenyl)-2, 3-dihydro-1H-indanone [(*R*)-TML104], a synthetic analog of resveratrol sesquiterpenes (±)-isopaucifloral F (Supplementary Figure 1A), on neointima formation.

## Materials and Methods

### Animal Model

Male C57BL/6J mice (25–30 g, 12 weeks, JOINN Lab, Suzhou, China) were maintained in a pathogen-free environment. Food and water were freely available under a controlled temperature (24 ± 1°C) with a 12/12 h dark/light cycle. We used a carotid artery injury mouse model, according to previously described protocols (2). Briefly, after mice were anesthetized with sodium pentobarbital (80 mg/kg, intraperitoneally), a midline neck incision was made, and the left carotid artery was exposed by blunt dissection. We then used blood vessel clamps to interrupt blood flow to the carotid arteries and made a lateral incision near the point of bifurcation of the external and internal carotid arteries. A guide wire (0.38 mm in diameter, NO.C-SF-15-15; Cook, Bloomington, USA) was inserted into the arterial lumen facing the aortic arch and rotated back and forth three times. After carefully removing the guide wire, the blood vessel was ligated at the lateral incision and the clamp was removed to restore blood flow. After vascular injury was induced, freshly prepared (*R*)-TML104 (10, 20 mg/kg) and atorvastatin (20 mg/kg) were administered daily by gastric gavage to the model group mice. (*R*)-TML104 and atorvastatin were both dissolved with saline. Mice were euthanized 28 days post-surgery by an overdose of sodium pentobarbital (150 mg/kg) via intraperitoneal injection.

### Antibodies and Reagents

Antibodies against α-smooth muscle actin (α-SMA, A11111) and β-Actin (AC026) were purchased from Abclonal (Wuhan, China). Antibodies against Ac-p65 (ab19870), NF-κB (ab16502), NOX1 (ab131088), NOX2 (ab129068), NOX4 (ab133303), proliferating cell nuclear antigen (PCNA, ab92552), cyclin D1 (ab134175), and SIRT1 (ab110304) were obtained from Abcam (Cambridge, UK). PDGF-BB was purchased from R&D (Minneapolis, USA). Goat anti-Mouse IgG (H+L) Cross-Adsorbed Secondary Antibody-Alexa Fluor 647 (A21235) and Goat anti-Rabbit IgG (H+L) Cross-Adsorbed Secondary Antibody-Alexa Fluor 555 (A21428) were purchased from Thermo Fisher Scientific (MA, USA). (*R*)-TML104 was synthesized and provided by Dr. Xun Sun's laboratory at the School of Pharmacy (Fudan University, China). Atorvastatin (MB1021) and resveratrol (MB1199) were purchased from Meilun Bio (Dalian, China). BAY 11-7082 (S1523) and N-acetyl-L-cysteine (S0077) was purchased from Beyotime (Shanghai, China). 4′,6-diamidino-2-phenylindole (DAPI) was purchased from Solarbio (Beijing, China).

### Histological and Morphometric Analysis

Fresh arteries samples were fixed in a 4% paraformaldehyde solution for 24 h and embedded in paraffin. The vascular tissue was cut into 5 μm sections, which were stained with hematoxylin and eosin (H&E) (G1120; Solarbio, China) for morphological analysis. Image-Pro Plus software (version 6.0, Media Cybernetics, MD, USA) was used to determine neointima formation. A mean value was generated from five independent sections of each artery sample.

### Immunofluorescence Staining

The 5 μm slices were cut from paraffin-embedded blocks and placed on microscope slides. Briefly, the sections were microwaved in the citric acid buffer to retrieve antigens for 30 min. Sections were then permeabilized with 0.1% Triton X-100 for 15 min and blocked with 1% bovine serum albumin for 30 min, incubated with primary antibody at 4°C overnight. The following antibodies were used: PCNA (1:100), cyclin D1 (1:100), α-SMA (1:100), SIRT1 (1:100). Afterward, sections were washed with PBS and incubated with appropriate secondary antibody (1:100 dilution; Alexa Fluor Plus 555) for 1 h at room temperature. Nuclei were then stained with DAPI. The images were obtained using a Zeiss LSM880 microscope (Zeiss, Gottingen, Germany). The integrated optical density values were obtained using the ImageJ Pro Plus software (version 6.0, Media Cybernetics).

### Cell Culture

Rat VSMC were enzymatically isolated from the Sprague-Dawley rats according to the protocols previously described (2). For functional studies, the cells were used between passages 3 and 5. VSMC were maintained in Dulbecco's Modified Eagle's Medium (DMEM, Hyclone, USA) supplemented with 10% fetal bovine serum (FBS, Hyclone, USA). Primary VSMC were maintained at 37°C under humidified 5% CO_2_/95% air atmosphere and their identity were confirmed using α-smooth muscle actin antibody.

### MTT Assay

The viability of VSMC was determined with 3-(4,5-Dimethyl-2-thiazolyl)-2, 5-diphenyl-2H-tetrazolium (MTT) bromide assay kit (C0009S; Beyotime Institute of Biotechnology, Shanghai, China). Briefly, the VSMC were plated in a 96-well microplate (5,000 cells/well). After the VSMC were incubated with different concentrations of (*R*)-TML104 for 24 h. Then, MTT reagent was added into the medium for a further 4 h. Next, the supernatant was then discarded, and dimethyl sulfoxide (DMSO) was added to solubilize the formazan crystals. The absorbance was measured at 570 nm with a microtiter plate reader (BIO-TEK, Winooski, VT, USA).

### EdU Assay

We used a 5-Ethynyl-2′-Deoxyuridine (EdU) incorporation assay (C0071S; Beyotime Institute of Biotechnology, Shanghai, China) to detect the proliferation of VSMC. Briefly, VSMC were seeded in 96-well plates. After growing to 60% confluence, the cells were serum-starved for 24 h. After the VSMC were incubated with different concentrations of (*R*)-TML104 for 4 h and subsequently treated with PDGF-BB for 24 h, and then incubated with EdU for 2 h. Next, the cells were fixed with 4% paraformaldehyde (P0099; Beyotime Institute of Biotechnology, Shanghai, China) for 30 min, permeabilized with 0.1% Triton X-100 for 10 min, and the cells were stained with Hoechst 33342 (50 μL/well) for 10 min. The images were captured using fluorescence microscopy (Nikon Eclipse Ti-S, Tokyo, Japan). The ratio of EdU-positive cells (EdU-stained cells/Hoechst-stained cells ×100%) was determined using a fluorescence microscope (Nikon Eclipse Ti-S, Tokyo, Japan).

### Cell Wound Assay

VSMC were seeded in a 6-well plate and scraped with a sterile tip in a straight line. The cells were immediately washed with cold phosphate buffer saline (PBS). After growing to 60% confluence, the cells were serum-starved for 24 h. After the VSMC were incubated with different concentrations of (*R*)-TML104 for 4 h and subsequently treated with PDGF-BB for 24 h. The images were taken by light microscopy (Olympus Optical Co, Tokyo, Japan). Wound healing images were analyzed using ImageJ Pro Plus software.

### Transwell Assay

The migration assay was performed using a transwell chamber (8 μm pore size, Corning costar, 3422, USA). Briefly, VSMC were seeded into each well of the upper chamber, and PBS or PDGF-BB were loaded into the bottom chamber. After growing to 60% confluence, the cells were serum-starved for 24 h. After the VSMC were incubated with different concentrations of (*R*)-TML104 for 4 h and subsequently treated with PDGF-BB for 18 h, the transwell membranes were fixed with 4% paraformaldehyde for 15 min. The membranes were stained with a 0.1% crystal violet solution for 10 min. The non-migrating cells on the top surface of the membrane were scraped with a cotton swab. Images were captured using light microscopy to quantify the average number of migrated cells. Five randomly chosen high-power fields (×200) in three independent experiments were used to calculate the average number of migrated cells. The migratory cells were evaluated by ImageJ Pro Plus software.

### Western Blot Analysis

VSMC were homogenized in lysis RIPA buffer on ice for 30 min and then centrifuged at 12,000 g for 15 min at 4°C. Protein concentrations were determined by using a BCA Protein Assay Kit (Cat.P0010; Beyotime Biotechnology, Shanghai, China). Equal amounts of protein were then separated in sodium dodecyl sulfate-polyacrylamide gel electrophoresis (SDS-PAGE) electrophoresis and then transferred onto polyvinylidene fluoride (PVDF) membranes. After being blocked with 5% skim milk plus tris-buffered saline for 1 h, the membrane was incubated with a primary antibody. The following antibodies were used: Ac-p65 (1:1000), NF-κB (1:2000), NOX1 (1:2000), NOX2 (1:2000), NOX4 (1:2000), PCNA (1:1000), cyclin D1 (1:1000), α-SMA (1:500), SIRT1 (1:1000), and β-Actin (1:10000) at 4°C overnight. The next day, the membrane was washed three times and then incubated with secondary antibodies (1:5000) for 1 h. Finally, the immunoreactive proteins were visualized using a chemiluminescence reagent (Millipore, Billerica, MA, USA). Signals were detected using a chemiluminescence system (Bio-Rad, Hercules, CA, USA). The β-Actin loading control was used for quantifying protein expression levels.

### ROS Detection and H_2_O_2_ Measurement

The dye, 2, 7-dichlorofluorescein diacetate (DCFH-DA, S0033S; Beyotime Institute of Biotechnology, China) was served as a fluorescence probe to detect intracellular ROS. Briefly, VSMC were incubated with DCFH-DA in a dark container at 37°C for 30 min. The cells were washed three times with PBS and finally analyzed using the FACSCalibu flow cytometry system (BD Biosciences, San Jose, CA, USA). The relative mean fluorescence intensity of each sample was analyzed using Flow Jo software version 10 (Tree Star Inc., Ashland, OR, USA). Intracellular H_2_O_2_ levels were detected using a Hydrogen Peroxide Assay Kit (S0038, Beyotime Institute of Biotechnology, Shanghai, China) according to the manufacturer's instructions. Briefly, scrape the lysed VSMC with a pipette tip and transfer cell lysate to a microcentrifuge tube. The cells were sufficiently homogenized and then centrifuged at 12 000 g for 5 min at 4°C. The supernatant was then incubated with a detection reagent for 30 min. The H_2_O_2_ production was assessed by using a microtiter plate reader.

### RNA Isolation and Quantitative Real-Time PCR

To determine the mRNA expression levels of genes, total RNA was isolated from VSMC using TRIzol reagent (Life Technologies, MA, USA), and cDNA was synthesized using a Prime Script RT reagent Kit according to the manufacturer's instructions. SYBR Green PCR reagents (Yeasen, Shanghai, China) were used to determine the relative expression of all gene transcripts by a Real-Time PCR Detection System (Applied Biosystems, Foster City, CA, USA). The expression of sample genes was quantified by the level of the β-Actin gene. The specific primers of NOX1, NOX2, NOX4, and β-Actin were available in Supplementary Table 1.

### Lentivirus Production and siRNA Transfection

The SIRT1 short hairpin was linearized plasmid and ligated into the pLVX vector. Lentivirus was produced by co-transfection of the SIRT1 lentiviral construct, the packaging plasmid psPAX2, and the envelope plasmid pMD2.G into HEK-293 T cells using Lipofectamine 3000 (Invitrogen, Carlsbad, CA, USA). The virus supernatant was harvested at 24 h and 48 h after transfection and stored at 4°C until the concentration step. The supernatant was filtered through a 0.45 μM filter and then centrifuged at 100,000 g for 2 h. The collected virus pellet was stored at −80°C. The mature antisense sequences of sh-SIRT1-1 and sh-SIRT1-2 were available in Supplementary Table 2. NOX4 or SIRT1 knockdown in VSMC was carried out by transfecting NOX4 or SIRT1 small interfering RNA (siRNA). The siRNA (20 nM) was transfected into VSMC using Lipofectamine 3000. All sequences of siRNAs were synthesized by Gene Pharma (Shanghai, China) and available in Supplementary Table 3.

### Statistics Analysis

Data were expressed as mean ± SD. Differences among three or more groups were determined using analysis of variance (ANOVA) followed by Tukey's *post-hoc* test. All statistical analyses were performed using GraphPad Prism (version 7.04; GraphPad Software Inc., San Francisco, CA, USA). Statistical significance was defined as ^*^p < 0.05, ^**^p < 0.01, ^***^p < 0.001.

## Results

### (*R*)-TML104 Mitigates Injury-Induced Neointima Formation *in vivo*

**Figure 1:**
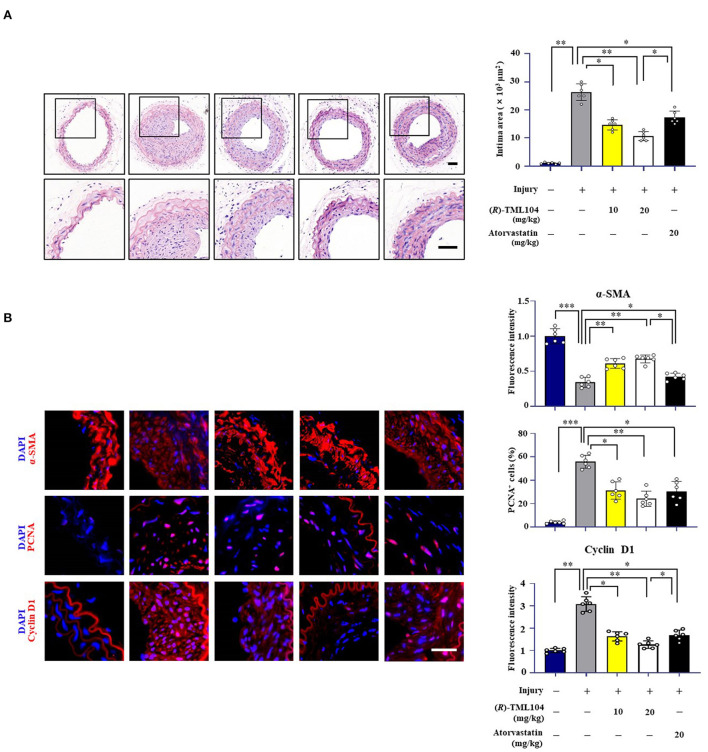
(*R*)-TML104 mitigates injury-induced neointima formation *in vivo*. **(A)** Hematoxylin and Eosin (H&E) staining of sections at 28 days after injury (Scale bar: 50 μm). **(B)** Immunofluorescence staining of α-SMA, PCNA, and cyclin D1 on sections of carotid arteries from mice. Scale bar: 50 μm, Data shown are means ± S.D (*n* = 6). *p < 0.05, **p < 0.01, ***p < 0.001.

To investigate whether (*R*)-TML104 affected injury-induced neointima formation, we treated the mice with two doses of (*R*)-TML104 (10, 20 mg/kg) after injury. (*R*)-TML104 treatment significantly decreased injury-induced neointimal area (Figure 1A). Meanwhile, (*R*)-TML104 treatment mitigated injury-induced downregulating α-SMA protein expression and upregulating PCNA and cyclin D1 expression (Figure 1B). Among the doses examined, (*R*)-TML104 at 20 mg/kg exhibited optimal protective effects and we used this dose for subsequent studies. To further confirm the function of (*R*)-TML104 on neointima formation, we used atorvastatin as a positive control. Interestingly, (*R*)-TML104 exhibits more prominent beneficial effects on neointima formation than atorvastatin at the same dosage (Figures 1A,B). Collectively, these findings demonstrate that (*R*)-TML104 could mitigate neointima formation *in vivo*.

### (*R*)-TML104 Inhibits PDGF-BB-Induced VSMC Phenotypic Transformation *in vitro*

**Figure 2:**
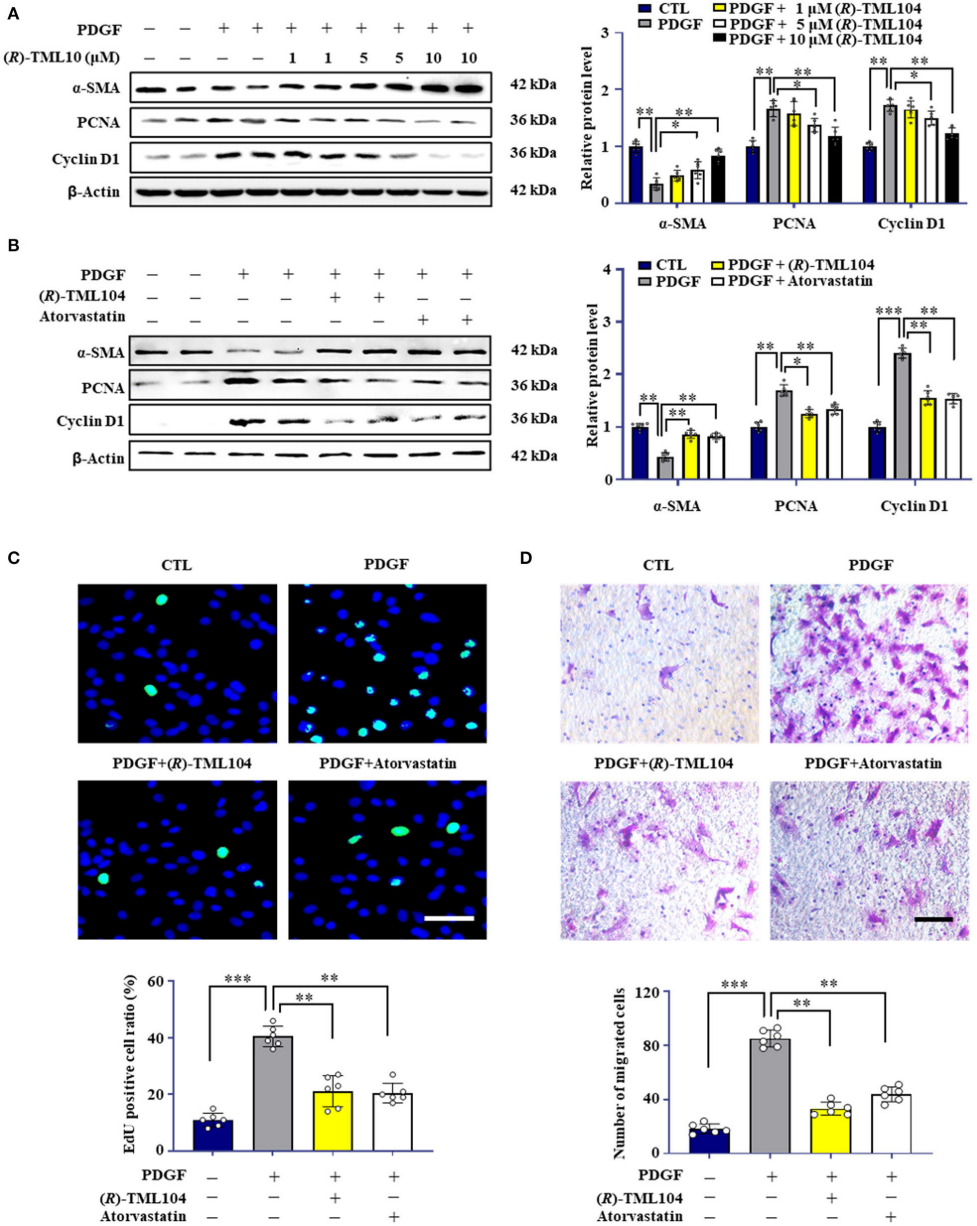
(*R*)-TML104 inhibits PDGF-BB-induced VSMC phenotypic transformation *in vitro*. **(A)** VSMC were pretreated with (*R*)-TML104 for 4 h and then stimulated with PDGF-BB (20 ng/mL) for 24 h. The protein levels of α-SMA, PCNA, and cyclin D1 were determined by western blotting. **(B)** The protein levels of α-SMA, PCNA, and cyclin D1 were determined by western blotting. **(C)** DNA synthesis in VSMC determined with EdU incorporation assay. Blue fluorescence (Hoechst 33342) showed cell nuclei and green fluorescence (EdU) stands for cells with DNA synthesis. **(D)** Transwell assay was performed to determine the migration of VSMC. Scale bar: 50 μm, Data shown are means ± S.D (*n* = 6). *p < 0.05, **p < 0.01, ***p < 0.001.

Phenotypic transformation of VSMC plays a vital role in neointima formation (27, 28). To investigate whether (*R*)-TML104 affects PDGF-BB-induced VSMC phenotypic transformation, we first detected the cytotoxicity of (*R*)-TML104 on VSMC. The MTT assay showed that (*R*)-TML104 (1-10 μM) had no significant effect on the viability of VSMC (Supplementary Figure 1B). As shown in Figure 2A, (*R*)-TML104 concentration-dependently reversed PDGF-BB-induced the expression of α-SMA, PCNA, and cyclin D1. Among the doses examined, (*R*)-TML104 at 10 μM exhibited optimal inhibitory effects yet no cytotoxic effect and we used this dose for subsequent studies. Meanwhile, the EdU assay showed that (*R*)-TML104 could inhibit PDGF-BB-mediated VSMC proliferation (Figure 2C). Followingly, the cell wound assay and transwell assay showed that (*R*)-TML104 could abolish PDGF-BB-induced VSMC migration (Figure 2D and Supplementary Figure 1C).

To further explore the effects of (*R*)-TML104 on VSMC phenotypic transformation, we chose atorvastatin and resveratrol as positive controls (29, 30). Notably, the protective effect of (*R*)-TML104 was similar to that of atorvastatin (Figures 2B–D and Supplementary Figure 1C). Moreover, we observed that resveratrol abolished PDGF-BB-induced the expression of PCNA and α-SMA (Supplementary Figure 1D). Interestingly, (*R*)-TML104 at the same dosage exhibited greater protective effects on these changes than resveratrol. Collectively, these results indicate that (*R*)-TML104 could inhibit PDGF-BB-induced VSMC phenotype transformation *in vitro*.

### (*R*)-TML104 Inhibits PDGF-BB-Induced VSMC Phenotypic Transformation by Upregulating SIRT1 *in vitro*

Resveratrol has beneficial effects on vascular disease by activating SIRT1 (31). SIRT1 has emerged as a critical target for VSMC phenotypic transformation (2, 12, 17). We hypothesized that (*R*)-TML104 inhibits PDGF-BB-mediated VSMC phenotypic transformation by modulating SIRT1. We then detected the expression of SIRT1 in VSMC in response to PDGF-BB. We found that SIRT1 expression was time-dependently and dose-dependently upregulated by (*R*)-TML104 treatment (Supplementary Figures 2A,B). Interestingly, (*R*)-TML104 exerted more significant effects on SIRT1 expression than resveratrol at the same dosage (Figure 2B).

**Figure 3:**
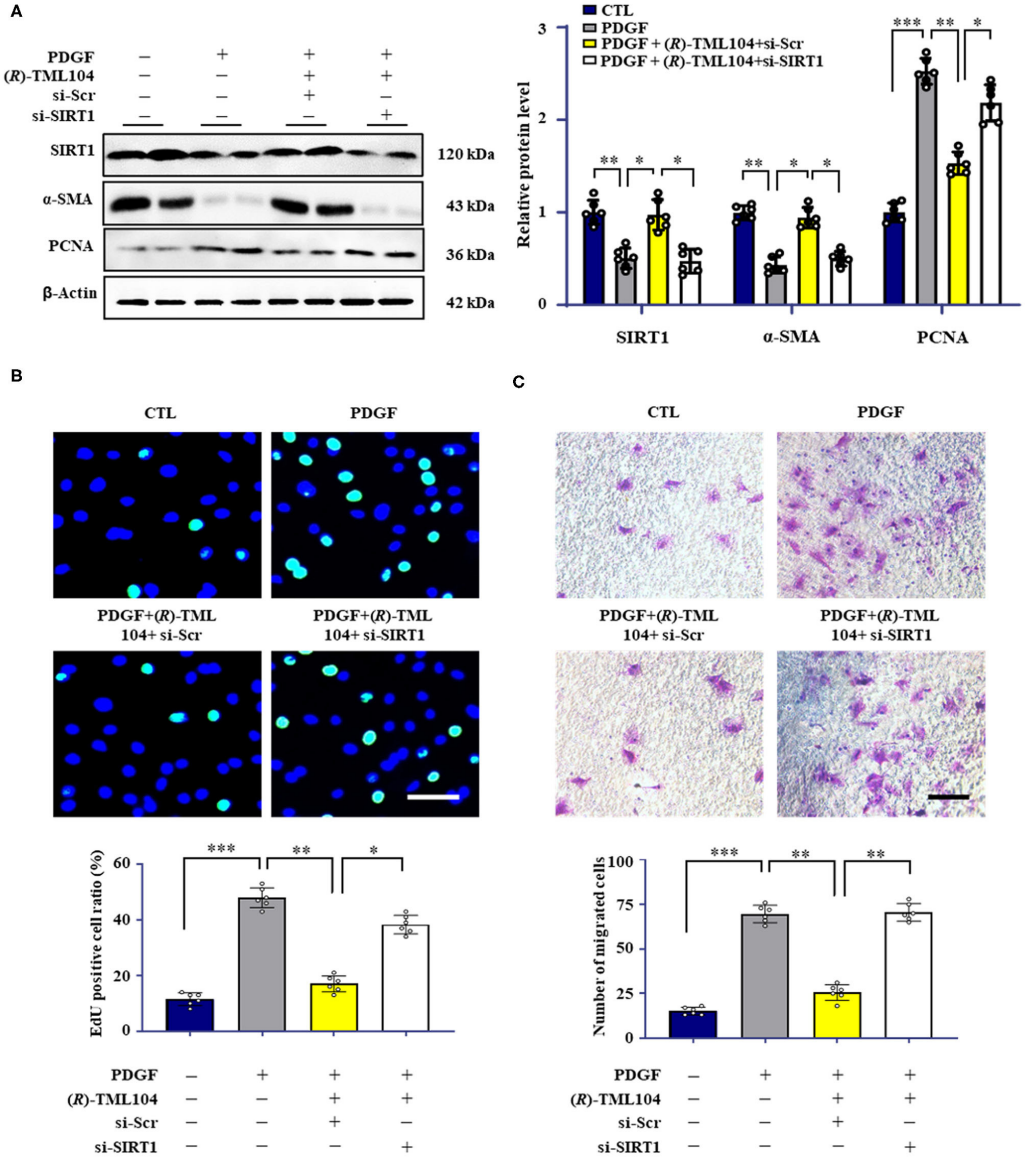
(*R*)-TML104 inhibits PDGF-BB-induced VSMC phenotypic transformation by upregulating SIRT1 *in vitro*. **(A)** VSMC were pre-treated with (*R*)-TML104 (10 μM) for 4 h and then stimulated with PDGF-BB (20 ng/mL) for 24 h. The SIRT1, α-SMA, and PCNA protein levels were determined by western blotting. **(B)** DNA synthesis was determined by the EdU incorporation assay. **(C)** VSMC migration was determined by transwell assay. Scale bar: 50 μm, Data shown are means ± S.D (*n* = 6). *p < 0.05, **p < 0.01, ***p < 0.001.

Our results showed that PDGF-BB decreased SIRT1 expression in VSMC, which is restored by (*R*)-TML104 treatment (Figure 3A). Next, we knocked down the expression of SIRT1 in VSMC by siRNA transfection. SIRT1 siRNA, but not control siRNA, markedly decreased (*R*)-TML104-mediated SIRT1 expression and abolished the inhibitory effects of (*R*)-TML104 on VSMC phenotypic transformation, as evidenced by increased PCNA expression (Figure 3A), decreased α-SMA expression (Figure 3A), increased EdU-positive (Figure 3B) and migrating cells (Figure 3C). Thus, our findings indicate that (*R*)-TML104 inhibits PDGF-BB-induced VSMC phenotypic transformation via upregulating SIRT1 *in vitro*.

### (*R*)-TML104 Mitigates Injury-Induced Neointima Formation by Upregulating SIRT1 *in vivo*

**Figure 4:**
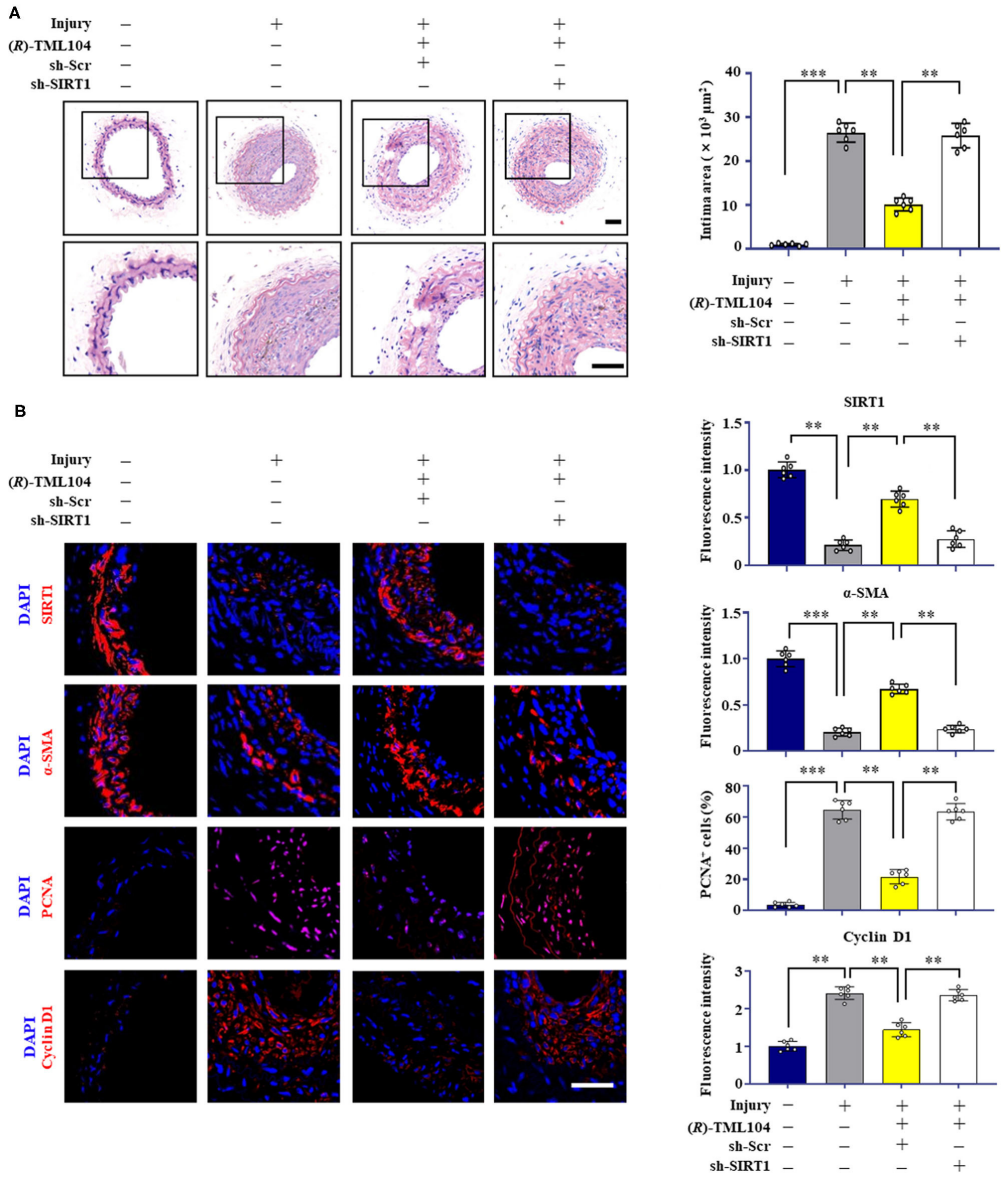
(*R*)-TML104 mitigates injury-induced neointima formation by upregulating SIRT1 *in vivo*. **(A)** After vascular injury, (*R*)-TML104 (20 mg/kg) was administered by gastric gavage to mice for 4 weeks. H&E staining of the sections of arterial neointima area. **(B)** Immunofluorescence staining of SIRT1, α-SMA, PCNA, and cyclin D1 on sections of carotid arteries from mice. Scale bar: 50 μm, Data shown are means ± S.D (*n* = 6) **p < 0.01, ***p < 0.001.

To investigate whether the inhibitory effects of (*R*)-TML104 on neointima formation were mediated by SIRT1 *in vivo*, we then investigated the expression of SIRT1 in mice. We found that SIRT1 expression was decreased in vascular tissue after injury, which was reversed by (*R*)-TML104 treatment (Figure 4B). Next, we delivered lentiviral shRNA to specific SIRT1 knockdown in mice. Lentiviral SIRT1 shRNA, but not control shRNA, markedly decreased (*R*)-TML104-mediated SIRT1 expression and significantly abolished the protective effect of (*R*)-TML104 on neointima formation (Figure 4A). Moreover, immunofluorescence staining showed that (*R*)-TML104-mediated PCNA, cyclin D1 and α-SMA expression was abolished by genetic SIRT1 knockdown (Figure 4B). These data demonstrate that (*R*)-TML104 inhibits neointima formation by upregulating the expression of SIRT1 *in vivo*.

### (*R*)-TML104 Inhibits PDGF-BB-Mediated VSMC Phenotypic Transformation by Modulating NOX4

**Figure 5:**
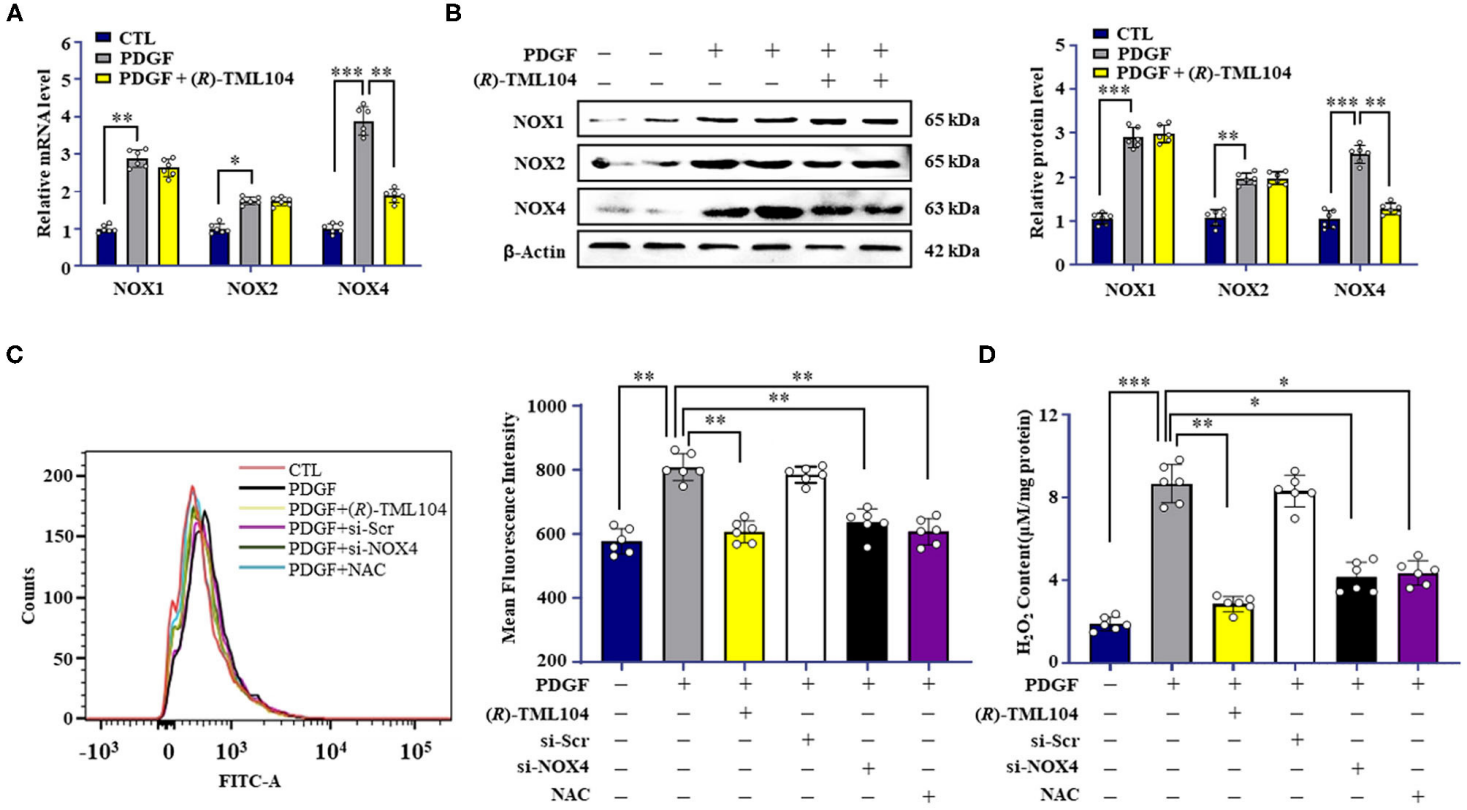
(*R*)-TML104 inhibits PDGF-BB-mediated VSMC phenotypic transformation by modulating NOX4. **(A)** VSMC were pretreated with (*R*)-TML104 (10 μM) for 4 h and then stimulated with PDGF-BB (20 ng/mL) for 6 h. Using real-time PCR, we measured NOX1, NOX2, NOX4 mRNA levels. **(B)** The NOX1, NOX2, and NOX4 protein levels were determined by western blotting. **(C)** ROS were quantitated by flow cytometry. **(D)** H_2_O_2_ concentrations were determined with a hydrogen peroxide assay. Data shown are means ± S.D (*n* = 6). *p < 0.05, **p < 0.01, ***p < 0.001.

It is reported that NOX-derived ROS plays a critical role in VSMC phenotypic transformation (32–34). In addition, SIRT1 can modulate the generation of ROS via regulating NOX expression (9, 35). To examine whether (*R*)-TML104 could modulate PDGF-BB-induced NOX expression in VSMC, the expression of NOX1, NOX2, and NOX4 in VSMC was measured. NOX1, NOX2, and NOX4 were all significantly higher both at the protein and mRNA level in VSMC in response to PDGF-BB when compared with control groups (Figures 5A,B). Intriguingly, (*R*)-TML104 treatment specifically inhibited the PDGF-BB-induced NOX4 expression, but not NOX1 or NOX2 expression both at the protein and mRNA level (Figures 5A,B). In addition, (*R*)-TML104 treatment also markedly reduced PDGF-BB-induced production of ROS (Figure 5C) and H_2_O_2_ (Figure 5D).

Next, we investigated the effect of NOX4 on VSMC phenotypic transformation, a NOX4-targeted siRNA was used to knock down the NOX4 expression. As expected, NOX4 siRNA, but not control siRNA, markedly decreased PDGF-BB-induced Nox4 expression in VSMC (Supplementary Figure 3A). PDGF-BB-induced the production of H_2_O_2_ (Figure 5C) and ROS (Figure 5D) was reduced by NOX4 siRNA. Moreover, NOX4 knockdown mimicked the inhibitory effects of (*R*)-TML104 on VSMC phenotypic transformation, as evidenced by decreased PCNA expression, increased α-SMA expression (Supplementary Figure 3A), reduced EdU-positive (Supplementary Figure 3B) and migrating cells (Supplementary Figures 3C,D).

To detect the role of ROS in PDGF-BB-induced VSMC phenotypic transformation, VSMC were treated with a ROS scavenger, N-acetyl-L-cysteine (NAC, 2 mM). Our results showed that NAC treatment significantly alleviated the PDGF-BB-increased ROS (Figure 5C) and H_2_O_2_ levels (Figure 5D). Meanwhile, NAC mimicked the inhibitory effects of (*R*)-TML104 on PDGF-BB-induced VSMC phenotypic transformation (Supplementary Figure 3A), proliferation (Supplementary Figure 3B) and migration (Supplementary Figures 3C,D). Collectively, these results suggest that (*R*)-TML104 inhibits PDGF-BB-induced VSMC phenotypic transformation through the NOX4-ROS signaling pathway.

### (*R*)-TML104 Regulates NOX4 by Modulating NF-κB Activation

**Figure 6:**
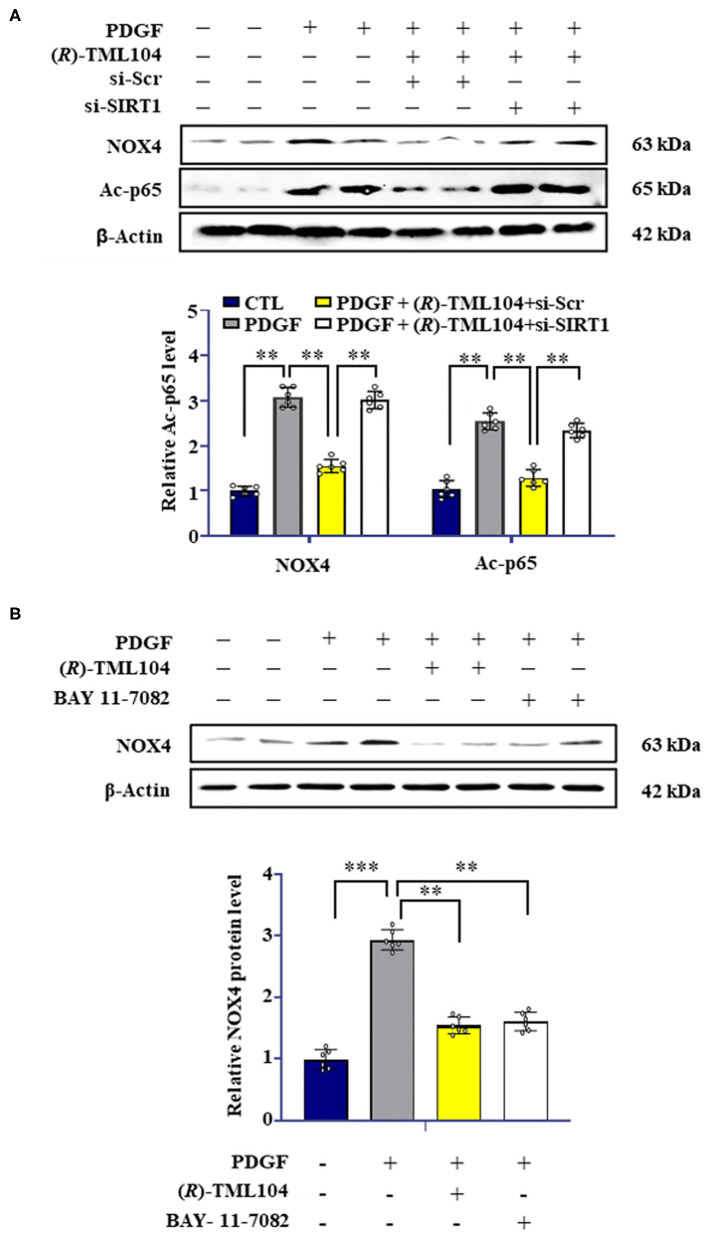
(*R*)-TML104 regulates NOX4 by modulating NF-κB activation. **(A)** VSMC were pretreated with (*R*)-TML104 (10 μM) for 4 h and then stimulated with PDGF-BB (20 ng/mL) for 4 h. The NOX4 and Ac-p65 protein levels were determined by western blotting. **(B)** The NOX4 protein levels were determined by western blotting. Data shown are means ± S.D (*n* = 6). **p < 0.01, ***p < 0.001.

Previous studies have shown that SIRT1 can regulate NOX4 expression (9, 36). We hypothesized that (*R*)-TML104-mediated NOX4 expression is regulated by SIRT1 in VSMC. Next, we measured the expression of NOX4 in VSMC by Western blot. It showed that SIRT1 knockdown by siRNA reversed (*R*)-TML104-mediated NOX4 expression in VSMC (Figure 6A). It is well-established that NF-κB activation is a crucial modulator of NOX4 expression (16, 37). In addition, NF-κB activation can be regulated by SIRT1-mediated deacetylation (38). Next, we investigated the status of NF-κB acetylation in VSMC. We found that NF-κB acetylation was increased in response to PDGF-BB, which is abolished by (*R*)-TML104 treatment. We then examined whether (*R*)-TML104-increased SIRT1 modulated NF-κB acetylation in VSMC. Next, we knocked down the expression of SIRT1 in VSMC by siRNA transfection. Our results showed that SIRT1 knockdown abolished the inhibitory effect of (*R*)-TML104 on the acetylation of NF-κB (Figure 6A).

To assess the role of NF-κB in NOX4 expression in VSMC, we used BAY 11-7082 (NF-κB inhibitor) to inhibit NF-κB activation. BAY 11-7082 treatment, similarly to (*R*)-TML104, suppressed PDGF-BB-induced NOX4 expression (Figure 6B). Collectively, these observations suggest that (*R*)-TML104-upregulated SIRT1 inhibits PDGF-BB-induced VSMC phenotypic transformation by downregulating NOX4 expression via decreasing NF-κB acetylation.

## Discussion

**Figure 7:**
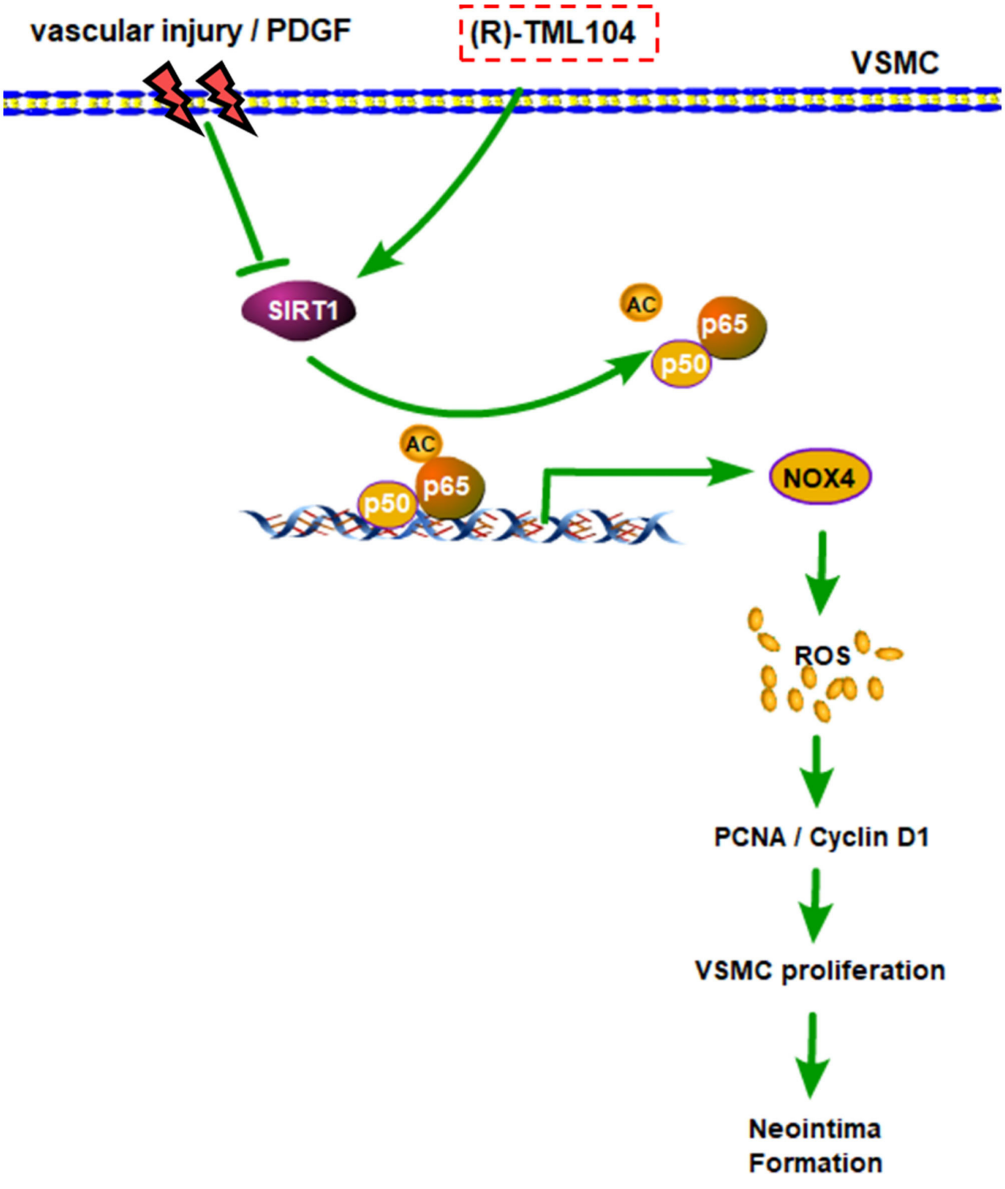
A schematic representation of modulatory effects of (*R*)-TML104 on neointima formation after injury. Our results showed that (*R*)-TML104 ameliorates neointima formation in a SIRT1-dependent mechanism. SIRT1 is decreased in VSMC by arterial injury *in vivo* or PDGF-BB *in vitro*. (*R*)-TML104 upregulates SIRT1 expression, and SIRT1 subsequently suppresses NOX4 expression by reducing NF-κB acetylation, thereby mitigates neointima formation. By this means, the treatment with (*R*)-TML104 could inhibit neointima formation in mice.

In the current study, we demonstrated that (*R*)-TML104 could prevent neointima formation *in vivo*. Furthermore, (*R*)-TML104 inhibited PDGF-BB-induced VSMC phenotypic transformation *in vitro*. We also found that SIRT1 expression is critical for (*R*)-TML104 to exert its protective effects. Finally, (*R*)-TML104 inhibited PDGF-BB-induced VSMC phenotypic transformation through NOX4 modulation via decreasing NF-κB acetylation. In summary, we found that (*R*)-TML104 against neointima formation and upregulates SIRT1 expression (Figure 7).

Previous work has demonstrated that resveratrol can protect from vascular disease (39). We hypothesized that (*R*)-TML104 could prevent vascular diseases, which was confirmed by our results showing that (*R*)-TML104 inhibited PDGF-BB-induced VSMC phenotypic transformation and injury-induced neointima formation. Next, we used atorvastatin as a positive control *in vivo* (40). Interestingly, the protective effects of (*R*)-TML104 against neointima formation were better than atorvastatin at the same dosage. We speculate that this superior effect of (*R*)-TML104 *in vivo* may be due to the key role of SIRT1, a well-known regulatory target of resveratrol, in the process of neointima formation (41). The expression of SIRT1 has been reported to decrease in neointima formation (2). In line with this observation, our data showed that SIRT1 decreases in VSMC in response to PDGF-BB, increased by (*R*)-TML104.

Increasing evidence has suggested that NOX4-derived ROS is crucial to the proliferation of several cell types (42, 43). A previous study showed that NOX4-derived ROS promote neointima formation (15, 44, 45). Consistently, our results showed that PDGF-BB increased NOX4-derived ROS levels, which was abolished by treatment with NOX4 siRNA or (*R*)-TML104. Consequently, we concluded that NOX4 down-regulation is responsible for the anti-oxidative effects of (*R*)-TML104 that confer vascular protection. In contrast, Chandrika showed that NOX4-derived ROS play an inhibitory role in the differentiation phenotypic of diabetic atherosclerosis (46). The diversity in NOX4-derived ROS functions may depend on specific environmental stimuli. Future work is needed to elucidate the complex role of NOX4-derived ROS in the development of vascular disease.

SIRT1 has been reported to regulate NOX4 expression in various biological processes (47, 48). Similarly, we found that (*R*)-TML104-increased SIRT1 inhibited PDGF-BB-induced NOX4 expression in VSMC, whereas SIRT1 knockdown abolished (*R*)-TML104-mediated inhibitory effects on NOX4 expression. Therefore, the fact that (*R*)-TML104 inhibits the PDGF-BB-induced expression of NOX4 likely depends on SIRT1 expression in VSMC. Previous studies have highlighted the influence of NF-κB-induced oxidative stress on the modulation of VSMC phenotypic transformation (30). In addition, NOX4 expression can be regulated by NF-κB activation (16). Hence, we evaluated whether (*R*)-TML104-reduced oxidative stress was associated with NF-κB activation. We found that (*R*)-TML104-increased SIRT1 inhibited NOX4 expression by reducing the acetylation status of NF-κB. This result is consistent with a previous report that SIRT1 regulated NOX4 expression by attenuating NF-κB acetylation in pancreatic cancer cachexia (9).

PDGF-BB is not the only factor that drives the injury-induced neointima formation (49, 50). A limitation in our study is that only PDGF-BB was used *in vitro* mechanistic study. The effect of (*R*)-TML104 on other factors-induced VSMC proliferation would be investigated as a follow-up study.

In summary, our data revealed that (*R*)-TML104-increased SIRT1 expression led to a reduction in NF-κB acetylation, thereby inhibit PPDGF-BB-induced VSMC phenotypic transformation by down-regulating NOX4 expression. Taken together, our findings suggest that (*R*)-TML104 may be an important therapeutic drug to prevent neointima formation.

## Data Availability Statement

The original contributions presented in the study are included in the article/Supplementary Material, further inquiries can be directed to the corresponding author/s.

## Ethics Statement

The animal study was reviewed and approved by Experimental Animal Center of Jiangnan University.

## Author Contributions

L-LP, XS, and JS designed the project. BY and HL performed the experiments. XD and XP performed the majority of the data analysis. L-LP and XD performed the final manuscript. All authors edited the manuscript.

## Funding

The work was supported by funds from the National Natural Science Foundation of China (Grant nos: 81973322, 82122068, 80270666, and 81870439), the Natural Science Foundation for Distinguished Young Scholars of Jiangsu Province (Grant no: BK20200026), Jiangsu Province Recruitment Plan for High-level, Innovative and Entrepreneurial Talents (Innovative Research Team), Wuxi Social Development Funds for International Science & Technology Cooperation (Grant no: WX0303B010518180007PB), Jiangsu Province “Six Summit Talents” program (Grant no: YY-038), Jiangsu Province Qing Lan Project, the Fundamental Research Funds for the Central Universities (Grant nos: JUSRP221037 and JUSRP22007), and Collaborative Innovation Center of Food Safety and Quality Control in Jiangsu Province and Wuxi Taihu Talent Project. Shanghai Municipal Committee of Science and Technology (Grant no: 17JC1400200).

## Conflict of Interest

The authors declare that the research was conducted in the absence of any commercial or financial relationships that could be construed as a potential conflict of interest.

## Publisher's Note

All claims expressed in this article are solely those of the authors and do not necessarily represent those of their affiliated organizations, or those of the publisher, the editors and the reviewers. Any product that may be evaluated in this article, or claim that may be made by its manufacturer, is not guaranteed or endorsed by the publisher.
