# Corrigendum: A novel resveratrol analog upregulates SIRT1 expression and ameliorates neointima formation

**DOI:** 10.3389/fcvm.2022.986353

**Published:** 2022-08-05

**Authors:** Baohui Yuan, He Liu, Xiaoliang Dong, Xiaohua Pan, Xun Sun, Jia Sun, Li-Long Pan

**Affiliations:** ^1^Wuxi School of Medicine and School of Food Science and Technology, Jiangnan University, Wuxi, China; ^2^State Key Laboratory of Food Science and Technology, Jiangnan University, Wuxi, China; ^3^School of Pharmacy, Fudan University, Shanghai, China

**Keywords:** (*R*)-TML104, neointima formation, nicotinamide adenine dinucleotide phosphate oxidase 4, nuclear factor-κB, vascular smooth muscle cells, reactive oxygen species, SIRT1

In the published article, there was an error in Figures 1, 2 as published. Due to our mistake in combining images, two graphs in Figures 1B, 2D were misused. The corrected Figures 1, 2 appear below.

**Figure 1:**
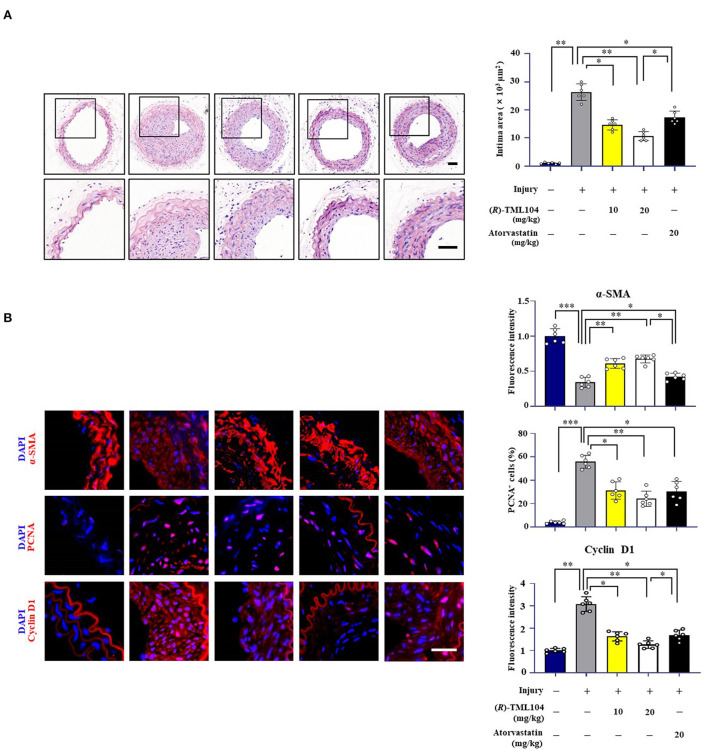
(*R*)-TML104 mitigates injury-induced neointima formation *in vivo*. **(A)** Hematoxylin and Eosin (H&E) staining of sections at 28 days after injury (Scale bar: 50 μm). **(B)** Immunofluorescence staining of α-SMA, PCNA, and cyclin D1 on sections of carotid arteries from mice. Scale bar: 50 μm, Data shown are means ± S.D (*n* = 6). *p < 0.05, **p < 0.01, ***p < 0.001.

**Figure 2:**
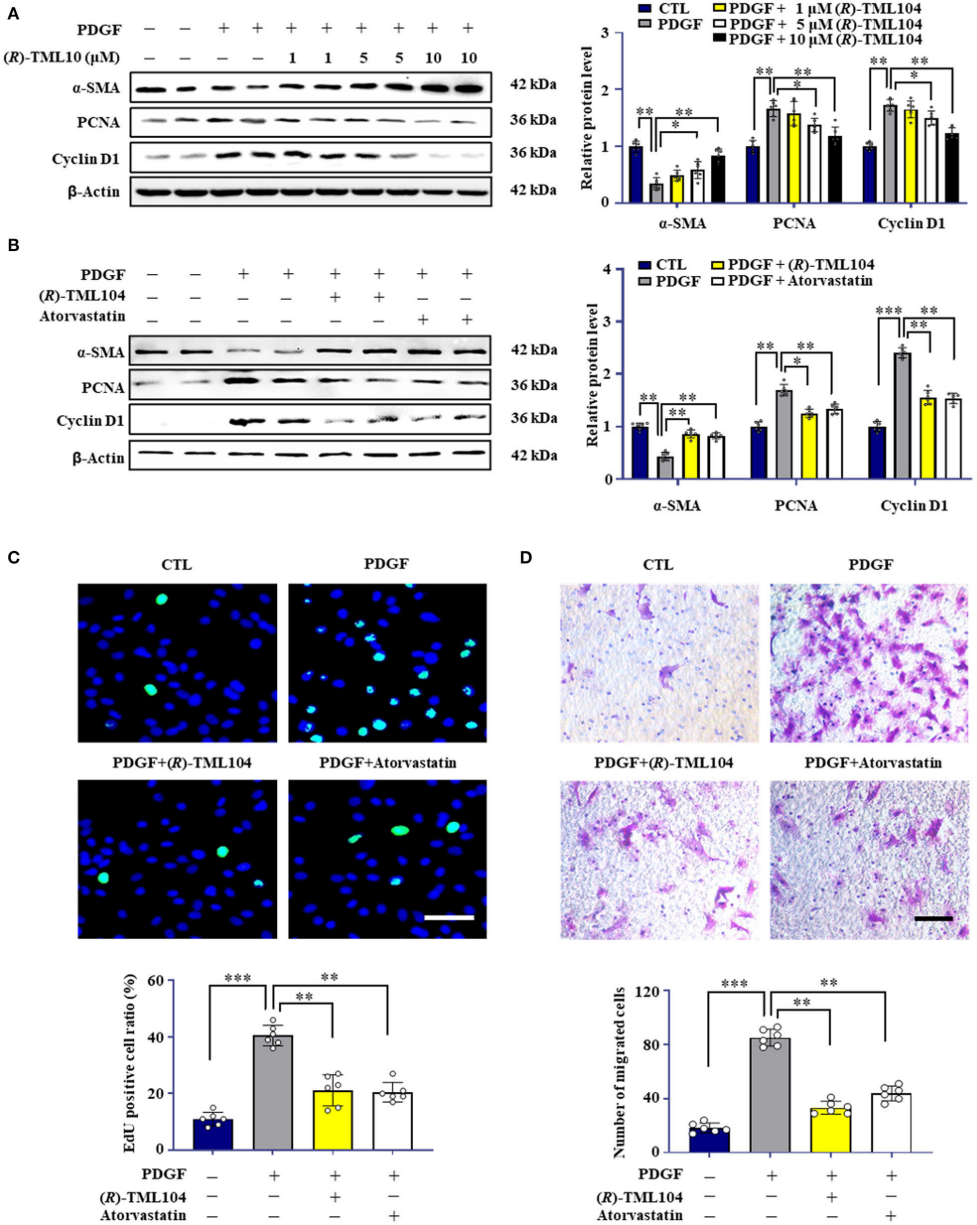
(*R*)-TML104 inhibits PDGF-BB-induced VSMC phenotypic transformation *in vitro*. **(A)** VSMC were pretreated with (*R*)-TML104 for 4 h and then stimulated with PDGF-BB (20 ng/mL) for 24 h. The protein levels of α-SMA, PCNA, and cyclin D1 were determined by western blotting. **(B)** The protein levels of α-SMA, PCNA, and cyclin D1 were determined by western blotting. **(C)** DNA synthesis in VSMC was determined with an EdU incorporation assay. Blue fluorescence (Hoechst 33342) showed cell nuclei and green fluorescence (EdU) stands for cells with DNA synthesis. **(D)** Transwell assay was performed to determine the migration of VSMC. Scale bar: 50 μm, Data shown are means ± S.D (*n* = 6). *p < 0.05, **p < 0.01, ***p < 0.001.

The authors apologize for this error and state that this does not change the scientific conclusions of the article in any way. The original article has been updated.

## Publisher's note

All claims expressed in this article are solely those of the authors and do not necessarily represent those of their affiliated organizations, or those of the publisher, the editors and the reviewers. Any product that may be evaluated in this article, or claim that may be made by its manufacturer, is not guaranteed or endorsed by the publisher.

