# Supplementary material for: A Novel Resveratrol Analog Upregulates SIRT1 Expression and Ameliorates Neointima Formation
Source: Front Cardiovasc Med. 2021 Nov 2;8:756098. doi: 10.3389/fcvm.2021.756098 (PMC8594564; doi:10.3389/fcvm.2021.756098)
Supplement: Supplementary file 1 [file Table_1.DOCX]

*Supplementary Material*

# Supplementary Figures and Tables

# 1. Supplementary Tables

# 1.1. Supplementary Tables S1. The Specific primers of NOX1, NOX2, NOX4

| Gene | 5' - 3' |
| --- | --- |
| NOX1(forward) | CTTCCTCAGTGGCTGGGATA |
| NOX1(reverse) | GCAAGCATTTGCGCAGGCT |
| NOX2(forward) | GTCGC CTATG GGATAGCTGTTAA |
| NOX2(reverse) | GGTAGCGTTCCAGGTTGAAGAA |
| NOX4(forward) | TGCCTGCTCATTTGGCTGT |
| NOX4(reverse) | CCGG CACATAGGTAAAAGGATG |
| β-Actin (forward) | GAGACCTTCAACACCC CAGCC |
| β-Actin (reverse) | TCGGGGCATCGGAACCGCTCA |

# 1.2. Supplementary Tables S2. The sequences of sh-SIRT1.

| Gene | 5' - 3' forward |
| --- | --- |
| SIRT1-shRNA#1 | CCGGGCCATGTTTGATATTGAGTATCTCGAGATACT  CAATATCAA ACATGGCTTTTTG |
| SIRT1-shRNA#2 | CCGGAGTGAGACCAGTAGCACTAATCTCGAGATTAG  TGCTACTGGTCTCACTTTTTTG |

# 1.3. Supplementary Tables S3. The sequences of si-RNA.

| Gene | 5' - 3' forward |
| --- | --- |
| SIRT1 siRNA | UGAAGUGCCUCAGAUAUUA |
| NOX4 siRNA | CCAGUGGUUUGCAGAUUUATT |
| Scrambled siRNA | CGUCCUG ACCUUUGAGUAUCU |

# 2. Supplementary Tables

## 2.1 Supplementary Figures 1


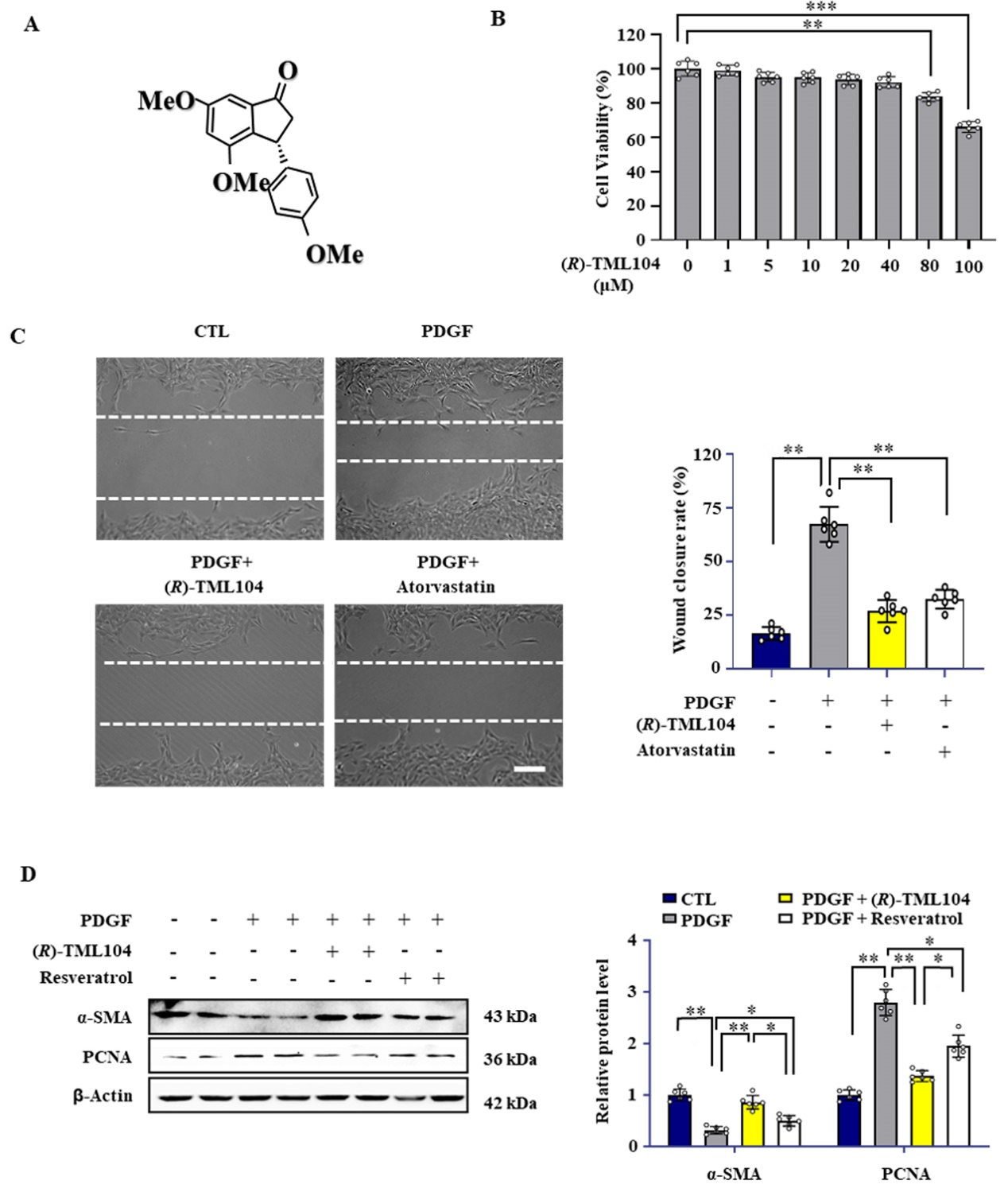


**Supplemental Figure 1**. (*R*)-TML104 inhibits the PDGF-BB-induced phenotypic transformation of VSMC. (**A**) The structure of (*R*)-TML104. (**B**) The viability of VSMC after 48 h incubation with (*R*)-TML104 (0, 1, 5, 10, 20, 40, 80 and 100 μM). (**C**) Cell wound assay was performed to determine the migration of VSMC. (**D**) The protein levels of α-SMA and PCNA were determined by western blotting. Scale bar: 50 μm, Data shown are means ± S.D, (n=6). * p < 0.05, * * p < 0.01, * * * p < 0.001.

## 2.2 Supplementary Figures 2


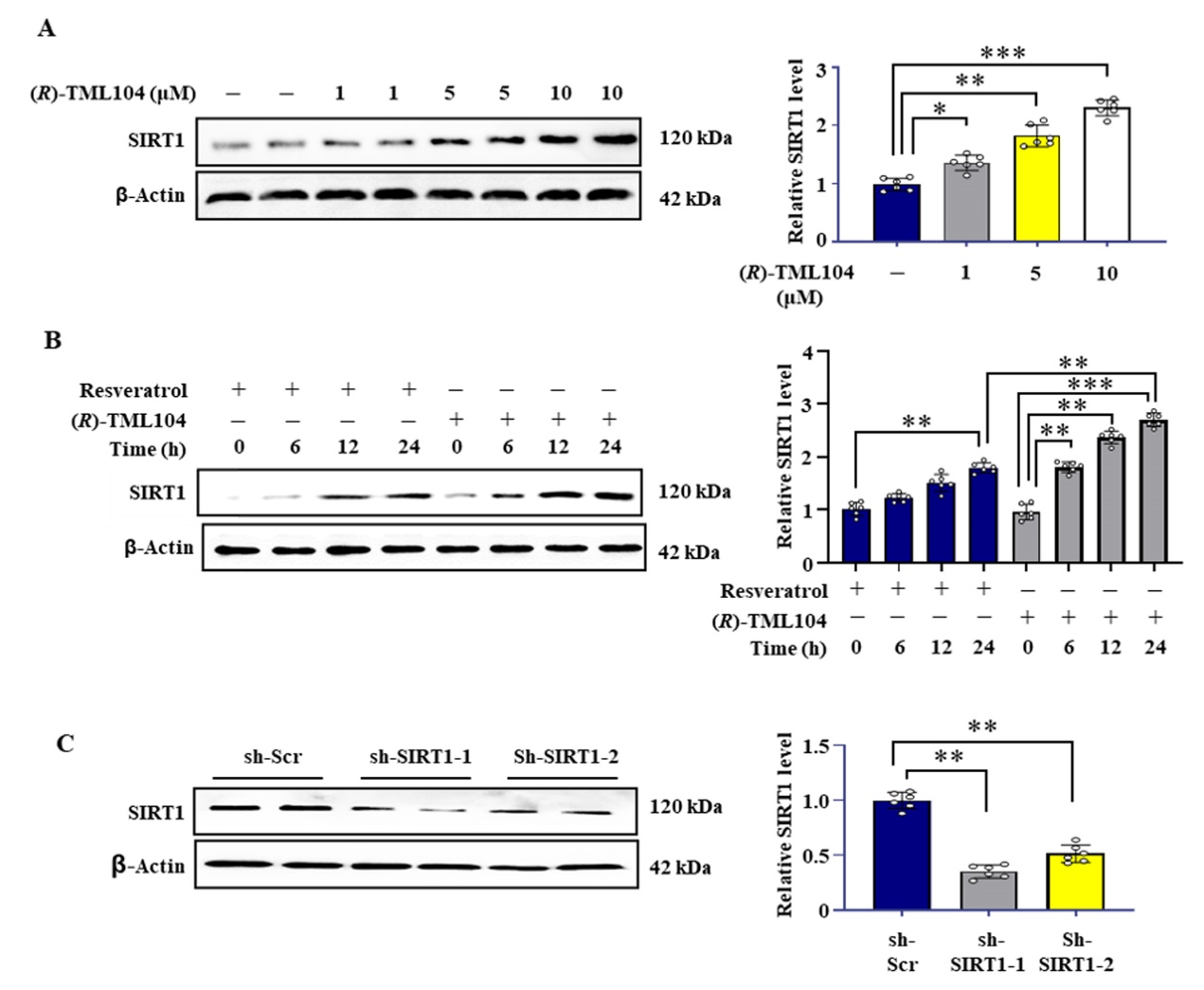


**Supplementary Figure 2.** (*R*)-TML104 upregulates the SIRT1 expression in VSMC. (**A**) The SIRT1 protein levels in VSMC treated with (*R*)-TML104 were determined by western blotting. (**B**) The SIRT1 protein levels in VSMC treated with (*R*)-TML104 or resveratrol were determined by western blotting. (**C**) The SIRT1 protein levels were determined by western blotting. Data shown are means ± S.D, (n=6). * p < 0.05, * * p < 0.01, * * * p < 0. 001.

## 2.3 Supplementary Figures 3


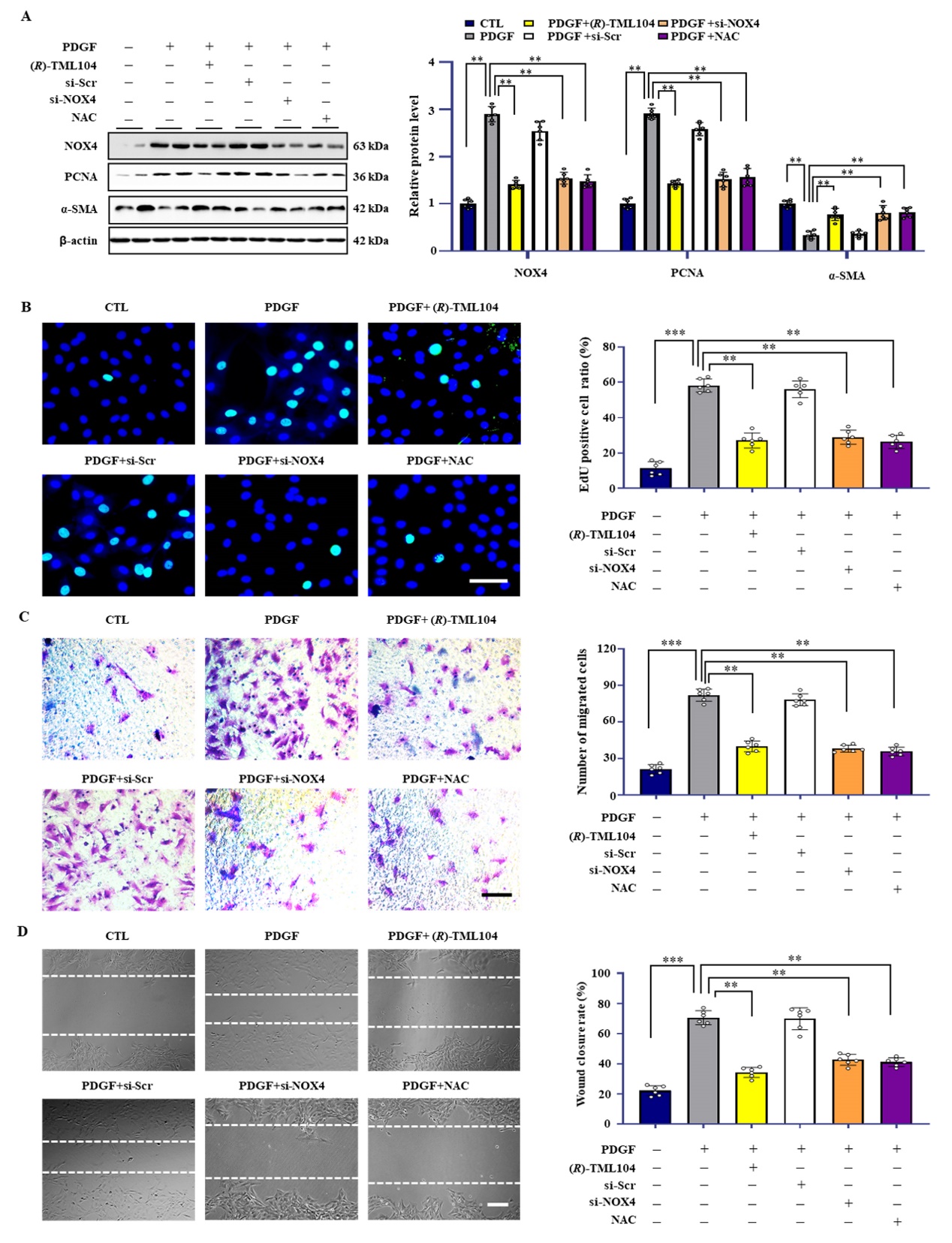


**Supplementary Figure 3.** (*R*)-TML104 inhibits PDGF-BB-induced VSMC phenotypic transformation via NOX4 inhibition. (**A**) The PCNA, α-SMA, and NOX4 protein levels were determined by western blotting. (**B**) DNA synthesis was determined by the EdU incorporation assay. (**C**) VSMC migration was determined by transwell assay. (**D**) VSMC migration was determined by cell wound assay. Scale bar: 50 μm, Data shown are means ± S.D, (n=6). * p < 0.05, * * p < 0.01, * * * p < 0. 001.
